# Studying and mitigating the effects of data drifts on ML model performance at the example of chemical toxicity data

**DOI:** 10.1038/s41598-022-09309-3

**Published:** 2022-05-04

**Authors:** Andrea Morger, Marina Garcia de Lomana, Ulf Norinder, Fredrik Svensson, Johannes Kirchmair, Miriam Mathea, Andrea Volkamer

**Affiliations:** 1 In Silico Toxicology and Structural Bioinformatics, Institute of Physiology, Charité Universitätsmedizin Berlin, Berlin, 10117 Germany; 2 BASF SE, 67056 Ludwigshafen, Germany; 3 Division of Pharmaceutical Chemistry, Department of Pharmaceutical Sciences, University of Vienna, Vienna, 1090 Austria; 4 Department of Pharmaceutical Biosciences, Uppsala University, Uppsala, 751 24 Sweden; 5 Dept Computer and Systems Sciences, Stockholm University, Kista, 164 07 Sweden; 6 MTM Research Centre, School of Science and Technology, 701 82 Örebro, Sweden; 7 Alzheimer’s Research UK UCL Drug Discovery Institute, London, WC1E 6BT UK

**Keywords:** Computational biology and bioinformatics, Drug discovery

## Abstract

Machine learning models are widely applied to predict molecular properties or the biological activity of small molecules on a specific protein. Models can be integrated in a conformal prediction (CP) framework which adds a calibration step to estimate the confidence of the predictions. CP models present the advantage of ensuring a predefined error rate under the assumption that test and calibration set are exchangeable. In cases where the test data have drifted away from the descriptor space of the training data, or where assay setups have changed, this assumption might not be fulfilled and the models are not guaranteed to be valid. In this study, the performance of internally valid CP models when applied to either newer time-split data or to external data was evaluated. In detail, temporal data drifts were analysed based on twelve datasets from the ChEMBL database. In addition, discrepancies between models trained on publicly-available data and applied to proprietary data for the liver toxicity and MNT in vivo endpoints were investigated. In most cases, a drastic decrease in the validity of the models was observed when applied to the time-split or external (holdout) test sets. To overcome the decrease in model validity, a strategy for updating the calibration set with data more similar to the holdout set was investigated. Updating the calibration set generally improved the validity, restoring it completely to its expected value in many cases. The restored validity is the first requisite for applying the CP models with confidence. However, the increased validity comes at the cost of a decrease in model efficiency, as more predictions are identified as inconclusive. This study presents a strategy to recalibrate CP models to mitigate the effects of data drifts. Updating the calibration sets without having to retrain the model has proven to be a useful approach to restore the validity of most models.

## Introduction

Machine learning (ML) models are usually trained—and evaluated—on available historical data, and then used to make predictions on prospective data. This strategy is often applied in the context of toxicological data to predict potential toxic effects of novel compounds^1–6^. Internal cross-validation (CV) is a common practice for assessing the performance of ML models. When applying the model to new data, it is advisable to observe the applicability domain (AD) of an ML model^7,8^. The AD determines the compound space and the response value (label) range in which the model makes reliable predictions^9^. Investigating classification models, Mathea et al.^8^ distinguished AD methods that rely on novelty from those relying on confidence estimation. Novelty detection methods focus on the fit of the query samples to the given descriptor space. Confidence estimation methods determine the reliability of the predictions by taking into account that samples may be well-embedded in the descriptor space but be unusual in terms of their class membership.

A popular method for confidence estimation is conformal prediction (CP)^10,11^. The framework of an inductive conformal predictor uses three types of datasets: proper training, calibration, and test set. The proper training set is used to train an underlying ML model. With this model, predictions are made for the calibration and test set. According to the rank that is obtained for the prediction outcome of the test compound as compared to the calibration set, so-called p-values are calculated to give an estimate of the likelihood of a compound to belong to a certain class. If a significance level, i.e. an expected error rate, is defined, the compounds are assigned labels for those classes where the p-value is larger than the significance level. For binary classification, the possible prediction sets are ‘empty’ ({$\emptyset$}), ‘single class’ ({0}, {1}), and ‘both’ ({0,1}). Single class predictions indicate a confident prediction for a certain class. Additionally, the CP framework recognises compounds for which it cannot make a reliable prediction ({$\emptyset$}) and compounds at the decision boundary, for which the predictions are reliable but indecisive ({0,1}). Provided that the calibration and test data are exchangeable, the framework of the conformal predictor is mathematically proven to yield valid predictions at a given significance level^10,11^.

The performance and AD of a model are determined by the quality and quantity of the data it has been trained on. One prerequisite for building good models is the availability of large, well-distributed and consistent datasets. To assemble large datasets, modellers often need to collect data from different sources, e.g. data which were produced in different assays or laboratories or over longer periods of time^12–14^. However, data from different sources and data taken at different time points may have distinct property distributions, reflecting, for example, the evolution of research interests or changes in assay technologies and protocols^15,16^. Since the predictivity of ML models is constrained by their AD, data drifts pose a challenge to modelling tasks, including toxicity or bioactivity prediction.

When ML models are validated using CV, the data is usually randomly split into training and test data. The resulting sets intrinsically stem from the same distribution and, typically, high model performance on the test set is observed. Nevertheless, it has been shown that model performance can be substantially lower for datasets obtained by time split or datasets from other sources^5,17–19^. This may be an indicator that the distribution of the data has changed. Hence, it is essential to confirm that ML models can be applied to a specific dataset and to determine the confidence in the predictions.

The data drifts, which challenge the underlying ML models, do also affect conformal predictors when the trained and calibrated models are applied to a new dataset. In previous work^17^, a new strategy was introduced to mitigate the effects related to data drifts by exchanging the calibration set with data closer to the holdout set. The study built on the Tox21 data challenge^2^, which was invented to support and compare ML models for twelve toxicity endpoints and included three subsequently released datasets. We showed that internally valid CP models resulted in poor performance when predicting the holdout data. The observed effects were associated to data drifts between datasets and could be mitigated by exchanging the calibration set with the intermediate set—without the need to retrain the models.

Here, we aim to expand and challenge our previous analysis on the recalibration strategy by a wider variety of datasets, beyond Tox21. Furthermore, we utilise enhanced compound encodings which combine molecular fingerprints with predicted bioactivity descriptors, specifically designed for toxicity prediction^12,20^.

First, temporal data drifts are studied using twelve toxicity-related endpoint datasets extracted from the ChEMBL database^21,22^. The ChEMBL database is a manually-curated data collection containing quantitative and qualitative measurements for more than two million compounds tested in up to more than 1.3 million assays. The large size of the database makes it a primary data resource for ML, in particular in the context of activity prediction^23–25^ and target prediction^26,27^. Moreover, it is one of only a few publicly-available bioactivity databases that provides temporal information on bioactivity measurements in the form of the publication date.

In the second part of this study, the impact on model validity from using data with differences in assay setups and source laboratories is investigated. Therefore, models were trained on public datasets for two in vivo endpoints, i.e., ‘liver toxicity’ and ‘in vivo micro nucleus test (MNT)’, and applied to predict proprietary data. Both, liver toxicity and MNT are in vivo endpoints with high relevance for the registration and authorisation of new chemical compounds^28–30^.

## Data and methods

In this section, first, the used datasets are described, including chemical structure standardisation, data splitting and compound encoding. Second, the CP setup together with the individual modelling strategies is explained. Finally, further data analysis and visualisation methods are outlined.

### Data assembly

#### Dataset description, collection and filtration

##### Large toxicity-related ChEMBL datasets

To investigate temporal data drifts, the ChEMBL database^21,22^ version 26 was queried following the protocol described by Škuta et al.^31^. In short, the presented 29 target datasets each containing more than 1000 compounds were downloaded with measured pIC50 values and publication year. Next, the datasets were cleaned to handle molecules contained more than once in a target dataset, called duplicates (see Supplementary Material Section A1.1). Then, compounds were standardised (see Section “Data assembly”) and the datasets temporally split (see Section “Data assembly”). Activity was assigned based on the target family and following the activity cutoff suggestions by the *Illuminating the Druggable Genome* Consortium^32^. Only datasets with more than 50 active and 50 inactive compounds in the holdout set were retained for the study. From the resulting 20 target datasets, only twelve targets that are linked to toxicity^33,34^ (see Supplementary Material Section A1.1 and Table 1) were selected for this study.

**Table 1:** ChEMBL target datasets used to investigate data drifts including the target name and the number of active and inactive compounds.

ChEMBL ID	Name	Active compounds	Inactive compounds
CHEMBL220	Acetylcholinesterase (human)	1334	1339
CHEMBL4078	Acetylcholinesterase (fish)	2056	1755
CHEMBL5763	Cholinesterase	1871	884
CHEMBL203	EGFR erbB1	2955	1104
CHEMBL206	Estrogen receptor alpha	826	590
CHEMBL279	VEGFR 2	3782	1392
CHEMBL230	Cyclooxygenase-2	1148	872
CHEMBL340	Cytochrome P450 3A4	2501	815
CHEMBL240	hERG	1601	3375
CHEMBL2039	Monoamine oxidase B	1413	1121
CHEMBL222	Norepinephrine transporter	406	1160
CHEMBL228	Serotonin transporter	449	1662

##### Public and inhouse datasets for liver toxicity and MNT

To assess drifts between data originating from different sources, public and proprietary datasets for two in vivo endpoints (drug-induced liver injury (DILI) and MNT) were collected. For CP model training, the same public datasets for DILI and MNT were used as compiled and described by Garcia de Lomana et al.^12^. After data pre-processing and deduplication the respective DILI dataset consists of 445 active and 247 inactive compounds; the MNT dataset of 316 active and 1475 inactive compounds (see Supplementary Material Section A1.2 for more details). Note that we will from here on refer to the DILI endpoint as ‘liver toxicity’.

Two proprietary BASF SE inhouse datasets for liver toxicity and MNT in vivo were used as independent test and update sets. In short, liver toxicity was measured in rats according to the OECD Guidelines 407, 408 and 422^35–37^. MNT was determined in mice following the OECD Guideline 474^29^, or in (non-GLP) screening assays. The liver toxicity dataset contains 63 active and 77 inactive compounds and the MNT dataset contains 194 active and 172 inactive compounds, after data pre-processing and deduplication (see Supplementary Material Section A1.3).

#### Chemical structure standardisation

Standardisation of chemical structures was conducted as described by Garcia de Lomana et al.^12^. Briefly, the SMILES of each of the compounds were standardised with the ChemAxon Standardizer^38^ node in KNIME^39,40^ to remove solvents and salts, annotate aromaticity, neutralise charges and mesomerise structures (i.e. taking the canonical resonant form of the molecules). Multi-component compounds as well as compounds containing any unwanted element were removed from the dataset. Canonical SMILES were derived for the standardised compounds and used for removing duplicates. In cases where duplicate SMILES had conflicting labels, the compounds were removed from the dataset.

#### Compound encoding

To encode the molecules for training the CP models, the ‘CHEMBIO’ descriptors developed by Garcia de Lomana et al.^12^ were used. These descriptors combine chemical with predicted bioactivity descriptors to describe the compounds. The chemical descriptor comprises a 2048-byte Morgan count fingerprint (with a radius of 2 bonds)^41^ and a 119-byte physicochemical property descriptor from RDKit^42^ (calculated with KNIME^39,40^).

For deriving the bioactivity descriptors, Garcia de Lomana et al.^12^ first built binary classification CP models for 373 in vitro toxicological endpoints, such as cytotoxicity, genotoxicity and thyroid hormone homeostasis (including datasets from ToxCast^33^, eMolTox^43^ and literature). These models were used to calculate the p-values (see Section “Conformal prediction”) per target endpoint model and class, thus, resulting in a 746-byte predicted bioactivity fingerprint. For use in CP-based toxicity prediction model studies, the individual features were scaled prior to model training. The combination of chemical and bioactivity descriptors into the 2913-byte ‘CHEMBIO’ descriptor has shown superior performance in the CP study by Garcia de Lomana et al.^12^ and was therefore used in this study.

#### Data splitting

After standardising the compounds (see Section “Data assembly”), the target datasets derived from the ChEMBL database were temporally split based on the publication year. This resulted in four subsets, i.e. train, update1, update2, and holdout set, see Table 2. Thus, compounds were ordered by publication year (old to new).

Aiming for the typically used ratio of 80% training (further divided in 70% proper training and 30% calibration set) and 20% test set^5,6,44^, year thresholds were set to assign at least 50% of the total compound number to the proper training set, and at least 12% to each calibration set. The remaining compounds were used as holdout data (see Supplementary Material Section A1.4 for more details).

For the computational experiments with the liver toxicity and MNT data, the standardised public datasets were used for training. The standardised proprietary data were time-split into update and holdout set based on the internal measurement date (see Supplementary Material Section A1.4 for details). Due to the small number of available inhouse compounds, only one update set was deducted, containing at least 50% of the total available inhouse dataset, see Table 2.

**Table 2:** Number of active and inactive compounds and year threshold used for the time split. ChEMBL data were temporally split into training, update1, update2 and holdout set based on the publication year. Models for the micro nucleus test and liver toxicity endpoint were trained on public data while the inhouse data were split into update and holdout set based on the internal measurement date.

Target (ID)	Training set			Update1 set			Update2 set			Holdout set		
	Thresh*	Inactive	Active	Thresh*	Inactive	Active	Thresh*	Inactive	Active	Thresh*	Inactive	Active
CHEMBL220	2014	802	840	2016	211	248	2017	217	138	2020	104	113
CHEMBL4078	2014	1031	1008	2015	259	275	2016	267	202	2020	499	270
CHEMBL5763	2015	1125	600	2016	302	75	2017	307	95	2020	137	114
CHEMBL203	2012	1660	433	2014	526	213	2016	428	291	2020	341	167
CHEMBL206	2006	437	325	2012	117	63	2016	114	97	2020	158	105
CHEMBL279	2010	1955	649	2013	523	307	2014	618	137	2020	686	299
CHEMBL230	2010	475	542	2013	218	78	2015	237	80	2020	218	172
CHEMBL340	2012	1272	496	2014	439	153	2015	341	59	2020	449	107
CHEMBL240	2012	797	1938	2014	301	413	2016	265	526	2020	238	498
CHEMBL2039	2014	710	645	2015	189	192	2017	380	212	2020	134	72
CHEMBL222	2009	231	673	2011	61	227	2015	40	206	2020	74	54
CHEMBL228	2009	242	858	2011	97	373	2014	31	235	2020	79	196
Micro nucleus test	-	1475	316	2005	70	134	–	–	–	2020	98	50
Liver toxicity	-	247	445	2011	42	48	–	–	–	2020	35	15

*Thresh: Data points published (ChEMBL) or measured (micro nucleus test, liver toxicity) until this year threshold are included in the corresponding subset.

### Conformal prediction

#### Inductive and aggregated conformal predictor

**Figure 1:**
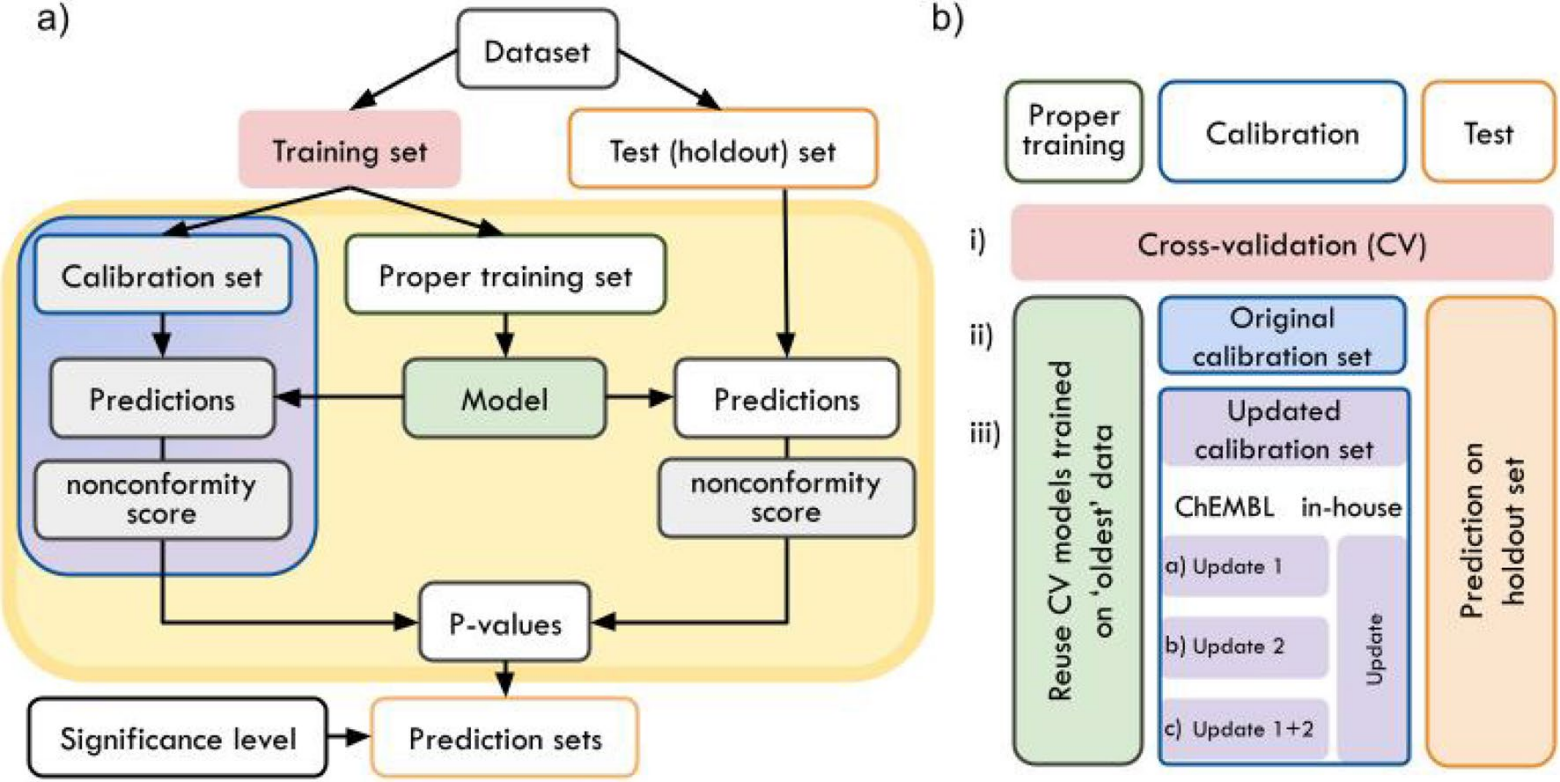
(**a**) Framework of an inductive conformal predictor. An ML model is fitted on the compounds of the proper training set to make predictions for the calibration and test (holdout) set instances. The predictions are transformed into nonconformity scores. By comparing the outcome of the test compound to the outcomes of the calibration set, p-values are calculated, which give an estimate on the likelihood of the compound to belong to a certain class. If a significance level is selected, prediction sets are calculated. *Blue-purple box* In the ‘update calibration set’ strategy, the calibration set is updated. *Yellow box* If multiple conformal predictors are aggregated, the part highlighted in the yellow box is repeated n times. (**b**) Overview of CP experiment setup: Experiments (i) CV, and prediction of holdout set using (ii) original calibration set, (iii) updated calibration sets to investigate temporal data drifts and drifts between data from different origin, i.e., ChEMBL and inhouse data.

The framework of an inductive conformal predictor (ICP) (see Fig. 1a) uses three types of datasets: proper training set, calibration set, and test set^45^. On the proper training set, an underlying ML model is fitted to make predictions for the calibration and test set instances. The outcomes, i.e. the probabilities for a compound to be assigned to class 0 or 1 in binary classification, are converted into so-called nonconformity (nc scores) by using a nonconformity function. Here, the inverse probability error function, which is typically used together with random forest (RF) models, is applied^20,46–48^.

For each test data point, the calibrated model outputs two so-called p-values in the binary setup. Therefore, the nc scores of the calibration set are sorted into two lists, one per class. The ratio of nc scores of the calibration set, which are larger than the nc scores for a test sample, results in a p-value. If a significance level, i.e. an expected error rate, is selected, prediction sets can be derived. They contain the class labels for which the p-value is larger than the significance level. For binary classification, the possible prediction sets are {$\emptyset$}, {0}, {1}, {0,1}. Given that calibration and test data are exchangeable, the CP framework ensures that the observed error rate does not exceed the significance level^10,11^.

In an ICP, only part of the information available in the training set is used for calibration as the other part is required to fit the underlying ML model. To improve the informational efficiency, multiple ICPs are typically aggregated in an aggregated conformal predictor (ACP)^47^, as in this study. Therefore, the training and prediction part (see yellow box in Fig. 1) is repeated n times (here $n=20$). In fact, the training set was 20 times split into calibration and proper training set, 20 models were built on the proper training set and calibrated with the corresponding calibration set. Each compound was predicted 20 times and the calculated p-values were aggregated taking the median value^49^.

#### Evaluation of conformal predictors

Conformal predictors are generally evaluated with respect to their validity, efficiency and accuracy of single class predictions. Validity is defined as the ratio of prediction sets containing the correct class label. As predictions are considered correct when they contain the correct label, ‘both’ predictions ({0,1}) are always correct. Empty prediction sets ({$\emptyset$}) count as erroneous. Efficiency of the predictions can be assessed by the ratio of prediction sets containing a single class label, i.e. {0} and {1}. The ratio of these single class predictions containing the correct label is often calculated as the single class accuracy. In the case of unbalanced datasets, class-wise metrics, i.e. separate metrics for the compounds belonging to the active and inactive class, can also be calculated. Balanced metrics (e.g. balanced validity, balanced efficiency and balanced accuracy), are then calculated as the arithmetic mean of the class-wise metrics.

#### CP setup and experiments

In this work, it was further explored how effects of data drifts can be mitigated by recalibrating a CP model. In the ‘update calibration set’ strategy, the original calibration set (Fig. 1a, blue-purple box) is exchanged with data assumed to be closer to the holdout set (Fig. 1b). Three main experiments were performed and compared. First, an internal fivefold CV experiment was performed (Fig. 1b.i). Hence, the training set was five times randomly stratified split into 80% training and 20% test set. Within each CV fold, an ACP consisting of 20 ICPs (inverse probability error function, Mondrian condition, nonconformist Python library, version 2.1.0^46^) using an underlying RF classifier (500 estimators, else default parameters, scikit-learn Python library, version 0.22.2^50^) was implemented. Each model was trained on 70% (proper training set) and calibrated on 30% (original calibration set) of the selected training data. The test sets from the CV-splits were predicted with the CV-models calibrated with the original training set. Second, the same calibrated CV-models were used to predict the holdout set, i.e. the ‘newest’ data from the ChEMBL datasets or the inhouse DILI and MNT test sets (Fig. 1b.ii). Third, the same models were recalibrated using the update sets, which were determined as described in Section “Data assembly”. For the experiments with the ChEMBL data, two update sets (update1 and update2) were used each, as well as a combination of update1+update2. For the inhouse data, only one update set was investigated. The recalibrated models were used to make predictions on the same holdout sets (Fig. 1b.iii) All models were evaluated at a significance level of 0.2, as it has been shown that this level offers a good trade-off between efficiency and validity^51,52^.

### Visualisation and further data analysis

#### Visualisation

Data visualisations were created using matplotlib version 3.2.1^53^.

#### UMAP

For descriptor space analysis, UMAPs were generated on the CHEMBIO fingerprints using the umap-learn Python library, version 0.4.6^54^. The parameters were set to $n\_neighbors = 100$, $min\_distances = 0.8$ and $distance\_metric = ``euclidean''$, meaning that a range of 100 nearest neighbours was considered to learn the manifold data structure. The distance between two points plotted in the UMAP is at least 0.8 and the distance between two data points is calculated using the euclidean distance.

#### Compound clustering

To analyse commonalities between compounds per set, compounds were clustered, using the “Hierarchical Clustering” node in KNIME. The clusters were annotated based on the Tanimoto coefficients of Morgan fingerprints (1024 bits, radius 2) between all compound pairs. A distance threshold of 0.5 was chosen, i.e., clusters were split so that all compounds within a cluster have a smallest distance below the threshold. Since the analysis focused on detecting clusters that spread over more than one set (training/test/update/holdout), clusters with less than two compounds, i.e. singletons, were not considered. Clustering and fingerprint calculation was performed in KNIME.

## Results and discussion

When using (ML) algorithms, it is assumed that the training data and test data are independent and identically distributed (*I*.*I*.*D*.). Similarly, CP models are designed to be valid if training and test data originate from the same distribution, i.e., are exchangeable^10^. This prerequisite, however, is not always fulfilled, especially when new compound spaces or different assay sources are explored. Hence, given comprehensive training data and modelling tasks, valid CP models can often be generated in a random-split k-fold CV setup. However, when predictions on external test data are performed, model performance has been shown to drop^55^. Here, we analysed the effects of data drifts on the validity of CP models. Thereby, we assessed the impact of recalibrating a CP model with updated data to restore the validity and positively affect performance. Note that this strategy has been introduced in the previous study, exemplified on the Tox21 challenge data^17^, and is further investigated here for different datasets, molecular encodings and study settings.

In the first part of this study, temporal data drifts were analysed on twelve toxicity-related datasets from the ChEMBL database. In the second part, the applicability of models trained on public data to proprietary toxicity datasets was investigated.

### Time-split experiments with twelve ChEMBL datasets

To analyse the impact of temporal data drifts on CP model performance, ChEMBL datasets for twelve endpoints were prepared. The selected endpoints are toxicologically-relevant targets, known for off-target effects, drug-drug interactions or as ecotoxicological endpoints, which need to be considered during the development of new chemicals^33,34^ (see Supplementary Table S1). The collected datasets were temporally split into training, update1, update2 and holdout subsets based on their publication date (see Section “Data and methods” and Table 2).

#### Experiments i and ii: CV and predictions using original calibration set

**Figure 2:**
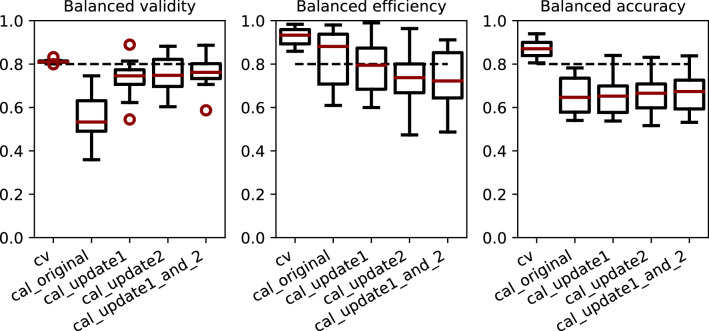
Time split evaluation (balanced validity, balanced efficiency, balanced accuracy) of CV experiments and predictions for the holdout set using the original (cal_original), update1 (cal_update1), update2 (cal_update2) and combined update1_and_2 (cal_update1_and_2) calibration sets for twelve ChEMBL datasets.

Fivefold CV on the training data produced valid (mean balanced validity: 0.81), efficient (mean balanced efficiency: 0.93), and accurate (mean balanced accuracy: 0.87) models at significance level of 0.2 (see experiment *cv_original* in Table 3 and Fig. 2). However, predictions with the same CV-models on the holdout data, i.e., newest data w.r.t. publication year, resulted in non-valid models with a higher-than-expected error rate (mean balanced validity of 0.56) as well as lower mean efficiency and accuracy (see experiment *cal_original* in Table 3 and Fig. 2). Class-wise evaluations for all experiments are provided in Supplementary Fig. S1.

The poor calibration of the model, i.e., a mean absolute loss in balanced validity of 0.25, for predictions on the holdout set may be an indicator for data drifts over time. Changes in the descriptor space or assay conditions (also due to diverse groups investigating the same target class) over the years may be responsible for such data drifts. Note that the data points in the holdout set were published at least five to ten years later than the training set instances (depending on the endpoint, see Table 2). Thus, it was investigated if the effects of these drifts can be mitigated by updating the calibration set with intermediately published data, i.e. update1 or update2 sets.

#### Experiment iii: update calibration set

To investigate whether valid models can be obtained with a small amount of new data, the calibration set was updated with more recent data while the trained CV-models were left unchanged^17^. For the ChEMBL experiments, the new calibration sets consist of the update1, update2 set, or a combination of both update sets.

Measured over all twelve endpoints, updating the calibration set with update1 or update2 led to an improvement of the mean balanced validity by up to 0.20 compared to the models with the original calibration set, reaching values of 0.73 and 0.76 with update1 and update2, respectively (see experiments *cal_update1* and *cal_update2* in Table 3 and Fig. 2). However, a slight decrease in the mean balanced efficiency by up to 0.09 was also observed (reaching values of 0.79 and 0.74 for update1 and update2, respectively).

It should be noted that restoring the validity is a prerequisite for applying CP models with confidence^7,17^. In the absence of validity, the confidence of the predictions is not guaranteed and the efficiency becomes an irrelevant metric (CP model would not offer any advantage and could be exchanged by the base model (e.g. random forest) to obtain an efficiency of one). With validity being a prerequisite for the application of CP models, restoring it by recalibration is an improvement. The concurrent loss in efficiency is undesired but also expected, since many instances in the holdout set may fall outside the AD of the underlying model. Lower efficiency along with improved validity indicates that the model recognises more compounds, for which it does not have enough information to classify them into a single class. Hence they are predicted as ‘both’. To avoid the loss in efficiency, the underlying model could be retrained with more up-to-date data. For example, compound representatives classified as empty or both sets by the current model could be experimentally screened to include their outcomes in an updated training set, feeding the model the necessary information to increase its efficiency. However, to achieve an improvement in the efficiency by retraining, a high amount of new data is usually required. Other studies^56–58^ have explored the use of CP-based active learning approaches to select data points that provide the most information to the model if experimentally evaluated. By using these approaches, a small number of additional data points can greatly extend the AD of the model.

While no overall improvement—or impairment—was observed in terms of accuracy (see Table 3 and Fig. 2), restored validity allows predictions with an associated confidence.

To analyse the impact of the size of the calibration set on the model performance, the two update sets were combined and used as a new calibration set (update1 + 2). In summary, all evaluation values remained at a similar level as for the update1 and update2 experiments. Mean balanced validity of 0.77, mean balanced efficiency of 0.73 and mean balanced accuracy of 0.67 were achieved (see experiment *cal_update1_and_2*) in Table 3 and Fig. 2). This indicates that the variation in size of the different calibration sets (from around 500 compounds in the original, update1, and update2 calibration sets to around 1000 compounds in the update1 + 2 set) in the ‘update calibration set’ strategy does not have a major influence on model performance in this study. Previous studies have shown that the size of the calibration set, nevertheless, has an influence on the resolution of the p-values, i.e. if more data points are available for calibration, the calculation of the p-values becomes more precise/distinct^6,17^. For instance, a calibration set with only 4 active compounds can only produce five different p-values, while a larger calibration set will be more precise in the p-value assignment.

**Table 3:** Overall, balanced and class-wise evaluation of time-split experiments with ChEMBL data.

	CV	Predict holdout set			
		Cal_original	Cal_update1	Cal_update2	Cal_update1_and_2
Validity	0.81 ± 0.01	0.57 ± 0.14	0.75 ± 0.07	0.77 ± 0.09	0.78 ± 0.07
Efficiency	0.93 ± 0.04	0.82 ± 0.14	0.78 ± 0.12	0.74 ± 0.13	0.73 ± 0.15
Accuracy	0.87 ± 0.04	0.68 ± 0.10	0.68 ± 0.08	0.70 ± 0.10	0.70 ± 0.09
Balanced validity	0.81 ± 0.01	0.56 ± 0.11	0.73 ± 0.09	0.76 ± 0.08	0.77 ± 0.08
Balanced efficiency	0.93 ± 0.04	0.83 ± 0.14	0.79 ± 0.12	0.74 ± 0.13	0.73 ± 0.15
Balanced accuracy	0.87 ± 0.04	0.65 ± 0.09	0.65 ± 0.09	0.66 ± 0.10	0.67 ± 0.09
Validity inactive class	0.81 ± 0.01	0.62 ± 0.26	0.76 ± 0.22	0.78 ± 0.22	0.78 ± 0.20
Efficiency inactive class	0.93 ± 0.04	0.84 ± 0.14	0.79 ± 0.14	0.72 ± 0.14	0.73 ± 0.16
Accuracy inactive class	0.87 ± 0.05	0.72 ± 0.26	0.69 ± 0.26	0.68 ± 0.29	0.70 ± 0.24
Validity active class	0.81 ± 0.01	0.50 ± 0.22	0.71 ± 0.19	0.74 ± 0.18	0.75 ± 0.14
Efficiency active class	0.93 ± 0.05	0.81 ± 0.14	0.78 ± 0.13	0.75 ± 0.10	0.73 ± 0.16
Accuracy active class	0.87 ± 0.04	0.59 ± 0.20	0.61 ± 0.26	0.64 ± 0.23	0.64 ± 0.20

#### ChEMBL data composition analysis

It is concluded that the validity of predictions for the holdout set can be restored when using more recent data to calibrate the CP models.

This could be attributed to the fact that the distribution of calibration and holdout sets are more similar compared to the training data. The efficiency of the models is slightly affected by this strategy, as the model still lacks information to make single class predictions. Nevertheless, the characteristics of the time-split within the ChEMBL data based on the publication year should be considered with care. In theory, a cluster CV (where by design compounds belonging to the same cluster are always in the same splits) should present a more challenging task than a temporal CV (where series of compounds could be further developed after the splitting date)^26^. However, this situation could be different for time splits on public domain data. Yang et al.^19^ showed on a benchmark study that time-split CV is a much harder task on public domain data (PDBbind^59–61^ in this case) than in industry setups. Using ChEMBL data, we observe that one publication may contain a whole chemical series, which was developed over a longer period of time, but is labelled in ChEMBL with the same publication date. Moreover, the fact that public data in ChEMBL arise from different sources reduces the chances that a compound series is further developed over time (and is therefore present in several splits). This might increase the chemical diversity between time-splits within openly collected data compared to data from a single institution. Analysing the molecular clusters of the ChEMBL data used in this study and their distribution among time-splits, we observed that only few clusters are scattered over different splits. Only between 7% and 16% of the compounds in a single cluster (with distance threshold of 0.5 and only considering clusters with at least two compounds) were spread over more than one split (see Supplementary. Fig. S5). This result indicates that, in this case, the prediction of the holdout set may be even more challenging than in an industrial (time-split) scenario, where early developed compounds of a compound series may be included in the consecutive training/update/holdout sets.

**Figure 3:**
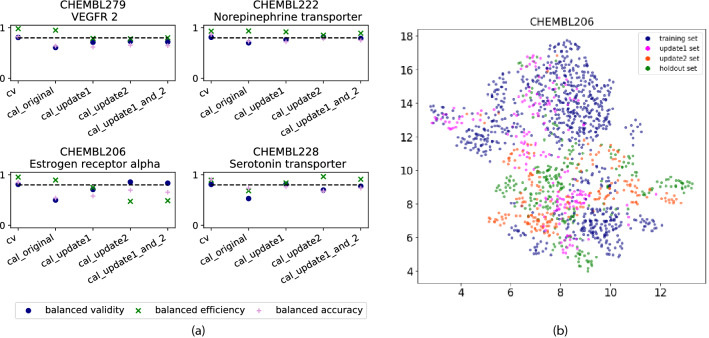
Analysis of individual endpoints (**a**) Balanced evaluation of time-split experiments for four selected ChEMBL endpoints. Each plot represents CV results (cv) and predictions for the holdout set using the original (cal_original), update1 (cal_update1), update2 (cal_update2) and combined update1_and_2 (cal_update1_and_2) calibration sets. The doted line at 0.8 denotes the expected validity for the chosen significance level of 0.2. (**b**) UMAP showing the descriptor space covered by the compounds in the different time-split sets for ChEMBL206 endpoint.

#### Individual endpoint performance analysis

The above discussed performance values referred to average values over models built for twelve endpoints. This led to the conclusion that updating the calibration set on average improves the validity at the cost of a small loss in efficiency. Considering the endpoints individually, the influence of updating the calibration set on the performance of the models varied. On average there was no substantial difference between updating the calibration set with update1 or update2 data. However, looking at individual models (Fig. 3a, Supplementary Fig. S4), e.g. endpoint ChEMBL228, the continuous calibration worked better in restoring the validity with update1 than update2 sets. In contrast, recalibrating with the update2 sets led to better performance for endpoints ChEMBL206, ChEMBL222, and ChEMBL279 (see also Supplementary Figs. S2 and S3).

The observations that the effects of recalibration for each endpoint are dependent on the update set might be explained by the descriptor space covered by the respective holdout, update and training sets. Our hypothesis is that updating the calibration set might be more beneficial if the update set compounds cover a descriptor space more similar to the holdout compounds than the original calibration set.

To investigate the influence of the descriptor space, the compounds’ ‘CHEMBIO’ descriptors of the training, update1, update2, and holdout set were transformed into a two-dimensional space using UMAP (Fig. 3b). For endpoint ChEMBL206, for which the update2 strategy worked clearly better, a large part of the update1 set overlaps with the training set, indicating that less improvement can be expected when recalibrating with it. Contrary, there is more overlap between the holdout and update2 sets. This might explain the particularly positive effects of recalibrating with update2 on the validity and accuracy for predicting the ChEMBL206 holdout set.

To quantify these differences in a rational manner, the Tanimoto coefficient based on Morgan fingerprints of each holdout compound to its nearest neighbour in the training and update sets, respectively, was calculated. Exemplified for endpoint ChEMBL206, the median coefficient of the holdout compounds to their nearest neighbour in the respective sets confirmed that the the holdout set is on average more similar to the update2 set (median coefficient of 0.42) than to the update1 or training sets (median coefficients of 0.29 and 0.33, respectively; distribution of distances to nearest neighbours provided in Supplementary Fig. S6).

### Update calibration strategy on inhouse datasets

When insufficient internal data are available to build ML models (or, in general, to extent the descriptor space coverage of the models), public data can be used in industrial setups for model training. Exemplified by MNT in vivo and liver toxicity CP models, we explored whether the applicability and validity of predictions on internal data could be improved by recalibrating models trained on public data with part of the internal data.

CP models were fitted on publicly-available data for MNT in vivo and liver toxicity, previously collected and used for model building by Garcia de Lomana et al.^12^. Liver toxicity induced by chemicals is a growing cause of acute liver failure^62^. MNT in vivo is an assay to assess mutagenicity^29^. Both endpoints are highly relevant for registration and authorisation of new chemicals^28–30^. The internal data were temporally split into update (older data) and holdout (more recent data) sets. Note that due to the limited data size only one update set was created (see Table 2).

#### Experiments i and ii: CV and predictions using original calibration set

The CP models were built on the publicly-available training data and validated within a fivefold CV. The predictions for the liver toxicity and the MNT endpoints resulted in a balanced validity of 0.81 and 0.82, a balanced efficiency of 0.81 and 0.79 and a balanced accuracy of 0.77 and 0.77, respectively (see Table 4). Thus, valid models with high efficiency and accuracy were obtained when evaluated within CV experiments.

Applying these models to the holdout set containing internal data, the balanced validity dropped drastically by up to 0.34 points (liver toxicity: 0.47, MNT: 0.50). The balanced accuracy of the models also decreased strongly (liver toxicity: 0.43, MNT: 0.49), while the balanced efficiency increased (liver toxicity: 0.89, MNT: 0.94). The latter indicates that mostly single class predictions were made. The class-wise evaluation of the MNT model predictions discloses that almost all internal compounds were predicted to be inactive (accuracy inactive compounds: 0.99, accuracy active compounds: 0, see Table 4 and Supplementary Fig. S7). For the liver endpoint, a similar trend was observed (accuracy inactive compounds: 0.7, accuracy active compounds: 0.16). These observations indicate that the distributions of the holdout and calibration data, i.e. of internal and external data, are highly different. Summarising, applying the models trained on public data to the internal data resulted in non-valid models that mainly predict all internal compounds as inactive.

**Table 4:** Evaluation of experiments to investigate drifts between internal and external data.

	Liver toxicity			Micro nucleus test		
	CV	Predict holdout set		CV	Predict holdout set	
		Cal_original	Cal_update		Cal_original	Cal_update
Balanced validity	0.81	0.47	0.82	0.82	0.50	0.74
Balanced efficiency	0.81	0.89	0.38	0.79	0.94	0.40
Balanced accuracy	0.77	0.43	0.49	0.77	0.49	0.39
Validity inactive class	0.81	0.75	0.84	0.80	0.99	0.61
Efficiency inactive class	0.84	0.84	0.45	0.79	0.89	0.54
Accuracy inactive class	0.77	0.70	0.63	0.75	0.99	0.29
Validity active class	0.82	0.20	0.80	0.83	0.00	0.88
Efficiency active class	0.78	0.95	0.31	0.79	1.00	0.26
Accuracy active class	0.77	0.16	0.35	0.78	0.00	0.50
Validity	0.82	0.58	0.84	0.81	0.66	0.70
Efficiency	0.80	0.87	0.40	0.79	0.93	0.45
Accuracy	0.77	0.52	0.57	0.76	0.63	0.33

#### Experiment iii: update calibration sets

**Figure 4:**
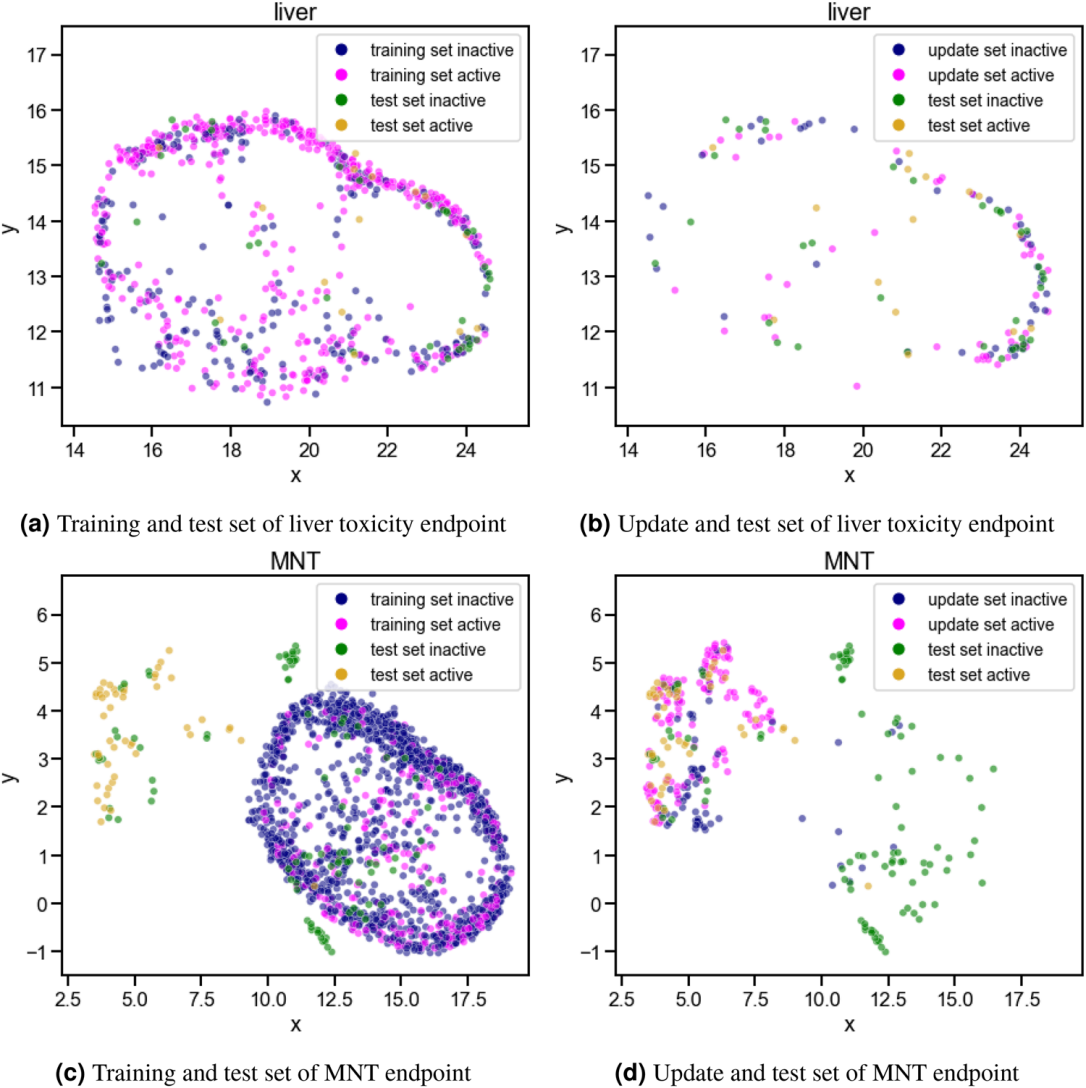
Descriptor space analysis of the liver toxicity (**a**, **b**) and MNT datasets (**c**, **d**) derived by UMAP. The descriptor space covered by the active and inactive compounds of the test sets is compared to the space covered by the training (**a**, **c**) and update sets (**b**, **d**), respectively.

For the liver toxicity endpoint, exchanging the calibration set with the earliest developed internal data (years 2005-2019, containing at least 50% of all internal data) could restore the validity for both compound classes (inactive: 0.84, active: 0.80). The balanced efficiency decreased largely from 0.89 to 0.38 (inactive compounds: 0.45, active compounds: 0.31) as many single class predictions were now identified as inconclusive and shifted to the ’both’ class. The balanced accuracy increased only slightly from 0.43 to 0.49. Nevertheless, the accuracy became more balanced (inactive: 0.63, active compounds: 0.35), as now more active compounds were correctly identified as such. The observations for the liver toxicity endpoint are similar to those for the ChEMBL endpoints. It is promising that the validity could be restored, although the balanced efficiency dropped. The improved balanced accuracy of 0.49 still leaves room for further improvements. To visualise the differences in the descriptor space covered by the public and internal data, UMAPs were derived (see Fig. 4a,b). Both datasets seem to cover a similar area of the descriptor space calculated with UMAP. The low accuracy obtained by applying the model on internal data could thus be better explained by the differences in the endpoint definition, as public and internal data were derived from different assays and species. These differences could lead to inconsistencies in the class labelling of a compound (i.e. one compound having different outcomes in each assay). Although the validity of the models could be restored by recalibration, these inconsistencies could be one explanation for the poor performance in terms of accuracy.

For MNT, updating the calibration set led to an improved balanced validity from 0.50 to 0.74 (inactive compounds: 0.61, active compounds: 0.88) and a strongly reduced balanced efficiency from 0.94 to 0.40 (inactive compounds: 0.54, active compounds: 0.26). The fact that the validity for the active class is high while the efficiency of this class remains low, indicates a high number of both predictions for the active compounds. Thus, the model is lacking information about active compounds to make single class predictions. A reduction in the balanced accuracy to 0.39 was observed, while the values are again more balanced between classes (inactive compounds: 0.29, active compounds: 0.50). Concluding, in the case of MNT, the balanced validity could be improved when recalibrating the models, but for the inactive compounds, it could not be restored to the expected level of 0.8. Analysing the descriptor space of the different datasets and their class labels (see UMAPs in Fig. 4c,d), it can be observed that almost all holdout compounds overlapping with the training set are inactive, while most of the holdout compounds overlapping with the update set are active. After updating the calibration set, the validity of the active class increased and could be restored, as this class is now better represented in the calibration set. However, the contrary is observed for the inactive class. Moreover, the efficiency drops as the analysed compounds are very different from the training set and the models are missing information about this area of the descriptor space to make single class predictions.

Although exchanging the calibration set with data from the same origin as the holdout set, i.e. with inhouse data, did help to increase the validity, these results show that the descriptor space of the holdout set still needs to be better represented by the training set to obtain efficient and accurate—and therefore useful—models.

## Conclusion

CP models, or generally ML models, are widely used for molecular property predictions, including activity and toxicity^5,6,63^. Notably, the CP framework is based on the assumption that test and calibration data stem from the same distribution^10,11^. If this prerequisite is not given, the models are not guaranteed to be valid (i.e. return the expected error rate). The goal of this study was twofold. Firstly, the performance of internally valid CP models, when applied to either newer time-split or (true) external data, was assessed. Second, the impact of model updating strategies exchanging the CP calibration set with data closer to the prediction set was evalutated. Building on previous work performed on the Tox21 datasets^17^, we investigated here two scenarios with data subsets that may stem from different distributions. First, temporal data drifts were analysed at the example of twelve toxicity-related datasets collected from the ChEMBL bioactivity database. Second, discrepancies between performance of models trained on publicly-available data vs. models recalibrated on inhouse data was evaluated on holdout inhouse data for the liver toxicity and MNT in vivo endpoints.

Due to changes in descriptor space and assays, over time or between laboratories, data drifts occur and were observed through the performed experiments (i and ii) on both the twelve ChEMBL as well as the liver toxicity and MNT datasets. Overall, valid CP models within CV were built for all endpoint datasets at a significance level of 0.2. In contrast, validity dropped below the expected error rate of 0.8, when applied to the holdout sets. Resulting mean balanced validities were 0.56 ± 0.11 over all twelve ChEMBL datasets, 0.47 for liver toxicity and 0.50 for MNT.

To address the poor validity on the holdout set, CP updating strategies were implemented (experiment iii), in which the calibration sets were exchanged by part of the newer or proprietary data, with the aim of restoring the validity. For most of the ChEMBL endpoints, the validity (at 0.2 significance level) could be mostly restored (mean balanced validity: 0.77 ± 0.08). The same holds for predictions on the proprietary liver toxicity endpoint data (balanced validity: 0.82). For the MNT data, the calibration was also improved, but to a lower extent (balanced validity: 0.74). Note that the improved validity comes at the cost of reduced efficiency for ten of the ChEMBL endpoints (average absolute loss between 0.04 and 0.10, depending on the update set used), which is more prominent for the liver toxicity and the MNT endpoints (absolute loss up to 0.55). A drop in efficiency is, however, more acceptable than non-valid models, which cannot be confidently applied. Too low efficiency may indicate that the model lacks information, e.g., chemical and biological descriptor space coverage, for classifying the new compounds.

With regard to the accuracy of the single class predictions, no change was observed on average for the ChEMBL endpoints when updating the calibration set. However, for the liver toxicity and MNT endpoints a more balanced accuracy between classes was observed after the update, as more compounds were identified as active.

In principle it is not possible to define an overall update/calibration criteria for all applications, but more research is needed to derive a generic approach on how to define it within the specific use-cases. In future studies it should be investigated how the degree of deviation of the calibration set from the training and holdout sets influences the models validity, efficiency and accuracy. This trade-off between the similarity of the calibration data to each set and the amount of available update data will probably determine in which scenarios the recalibration strategy is a good approach to overcome data drifts, and when a complete model retraining is necessary.

It is in the nature of the field of compound toxicity prediction or drug design that ML models are applied to completely new compounds that are potentially quite different from the training set. This work showed the necessity of considering data drifts when applying CP or ML models to new and external data and the need of developing strategies to mitigate the impact on the performance.

## Supplementary Information

Supplementary Information.

## Data Availability

The input data for the twelve ChEMBL endpoint models can be retrieved from https://doi.org/10.5281/zenodo.5167636. The public data for the liver toxicity and in vivo MNT endpoints are freely available as described in Garcia de Lomana et al.^12^. The in house data for liver toxicity and in vivo MNT are proprietary to BASF SE.

## Code availability

Code is available on GitHub at https://github.com/volkamerlab/CPrecalibration_manuscript_SI. The GitHub repository contains example notebooks on how to perform the recalibration experiments on a selected endpoint as well as on all twelve ChEMBL endpoints together. The code can be adapted and used for other datasets.
