# Supplementary material for: Studying and mitigating the effects of data drifts on ML model performance at the example of chemical toxicity data
Source: Sci Rep. 2022 May 4;12:7244. doi: 10.1038/s41598-022-09309-3 (PMC9068909; doi:10.1038/s41598-022-09309-3)
Supplement: Supplementary file 1 — Supplementary Information. [file 41598_2022_9309_MOESM1_ESM.pdf]

Additional file

Studying and mitigating the effects of data drifts on ML model performance at the example of chemical toxicity data

Andrea Morger, Marina Garcia de Lomana Rodriguez, Ulf Norinder, Fredrik Svensson, Johannes Kirchmair, Miriam Mathea and Andrea Volkamer

A1 Additional information on data and methods

A1.1 Target selection for the ChEMBL datasets

Target datasets were selected following a collection of 1360 ligand sets provided by Škuta et al.[31] for similarity searching, bioactivity classification and scaffold hopping. First, the 29 target datasets, for which Škuta et al. found $\geq$ 1000 compounds with reported pIC50 values, were downloaded, including pIC50 values and publication year. The following cleaning procedure was applied to each target dataset: If there were multiple measurements per compound and endpoint, the mean and standard deviation were calculated. Only the mean measurement of those duplicates was kept if the standard deviation was lower or equal than 0.5, otherwise they were discarded. The oldest publication year (i.e. lowest number) was kept for aggregated data points. The compounds were standardised as described in the main manuscript (section 2.1.2) and temporally split into training, update1, update2, and holdout set as explained in 2.1.4. If fewer than 50 active and 50 inactive compounds were left in the holdout set after the time-split, the target dataset was excluded from the study. Finally, 20 targets remained which match the filtering criteria. Of these, a total of twelve targets were selected that are linked to toxicity. A target was defined to be associated to toxicity if it was either assayed in ToxCast[33], or part of the list of targets that are recommended to early assess the potential hazard of a compound[34].

A1.2 Public datasets for liver toxicity and MNT

To assess drifts between data originating from different sources, public and proprietary datasets for liver toxicity and micro nucleus test (MNT) were collected. For CP model training, the same public datasets for liver toxicity (more specifically here drug-induced liver injury (DILI)) and MNT in vivo were used as described by Garcia de Lomana et al.[12]. Data for the DILI endpoint were gathered from the U.S. Food and Drug Administration (FDA)[?] and for the MNT in vivo endpoint from three sources (eChemPortal[?], the work of Benigni et al.[?] and Yoo et al.[?]). The respective datasets contain 692 (445 active and 247 inactive compounds) and 1791 compounds (316 active and 1475 inactive compounds) after the data pre-processing and deduplication steps conducted by Garcia de Lomana et al.[12].

A1.3 Inhouse datasets for liver toxicity and MNT

Two inhouse datasets for liver toxicity and MNT in vivo, with data generated by BASF SE, were used as holdout and update set to investigate data drifts between data with different origin. Liver toxicity was measured in oral assays on rats (including OECD Guidelines 407, 408 and 422, as well as range finding oral studies). Compounds showing adverse or adaptive effects in the liver in any of these studies were labelled as active. MNT in vivo was determined in mice in an assay following the OECD Guideline 474 or in (non-GLP) screening assays (with 18 animals). The liver toxicity dataset contains 140 (63 active and 77 inactive) compounds and the MNT in vivo dataset contains 366 (194 active and 172 inactive) compounds after the data pre-processing and deduplication steps (following the same procedure as Garcia de Lomana et al.[12], see "Chemical structure standardisation").

A1.4 Time-splitting procedure

Note that all compounds published (ChEMBL data) or assayed (inhouse data) in the same year were assigned to the same split.

ChEMBL data After standardising the compounds (see 2.1.2), the ChEMBL data were time-split into four datasets, i.e. train, update1, update2, and holdout set based on the publication year. A minimum number of compounds per dataset was defined based on a predefined ratio, i.e. the training set must contain at least 50% of the total number of compounds, the update1 and update2 sets must contain at least 12% each. Starting from the earliest year, all compounds published in that year were assigned to the training set and the number of training compounds was assessed. Same for the next year(s) until the training set contained at least the minimum number of training compounds defined. Then, all compounds published in the following year(s) were assigned to the update1 set until the respective threshold was reached. With the same procedure, the compounds published in the subsequent year(s) were allocated to the update2 set. All remaining compounds belong to the holdout set. The number of active and inactive compounds available per subset of the twelve holdout ChEMBL target datasets, as well as the corresponding time thresholds for splitting, are provided in Table 2.

Liver toxicity and MNT data To investigate the occurrence of discrepancies between external and internal data (see A1.2), the liver toxicity and MNT datasets were investigated. The external data were used for model building as well as for the original calibration set. The internal data were time-split into update and holdout set based on the date they were measured internally. Due to the small number of available inhouse compounds, only one update set was deducted. The data was selected by year as described for the ChEMBL data until at least 50% of the compounds were assigned to the update set. The number of training, update and holdout compounds available for the liver toxicity and MNT endpoints are shown in Table 2.

Table S1. ChEMBL datasets and their biological relevance. A selection of possible toxicological or adverse effects due to agonism (or activation) or antagonism (or inhibition) with the targets is provided.

ChEMBL ID	name	toxicological or adverse effects
CHEMBL220	Acetylcholinesterase (human)	decreased blood pressure or heart rate, increased GI motility[?,34]
CHEMBL4078	Acetylcholinesterase (fish)	decreased blood pressure or heart rate, increased GI motility[?,34]
CHEMBL5763	Cholinesterase	decreased heart rate, QT interval prolongation[?]
CHEMBL203	EGFR erbB1	skin toxicity, cardiotoxicity[?,?]
CHEMBL206	Estrogen receptor alpha	antiandrogenic effects, hormone-dependent cancers[?,?]
CHEMBL279	VEGFR 2	hypertension, disturbed wound healing, GI and skin toxicity[?]
CHEMBL230	Cyclooxygenase-2	myocardial infarction, increased blood pressure, ischaemic stroke, atherothrombosi[?,34]
CHEMBL340	Cytochrome P450 3A4	drug-drug interactions, detoxification by metabolism, activation of toxic metabolites[?]
CHEMBL240	HERG	QT interval prolongation[?]
CHEMBL2039	Monoamine oxidase B	cell death[?]
CHEMBL222	Norepinephrine transporter	increased heart rate or blood pressure, constipation[?,34]
CHEMBL228	Serotonin transporter	increased GI motility, insomnia, anxiety, sexual dysfunction[?,34]

A2 Additional information on results

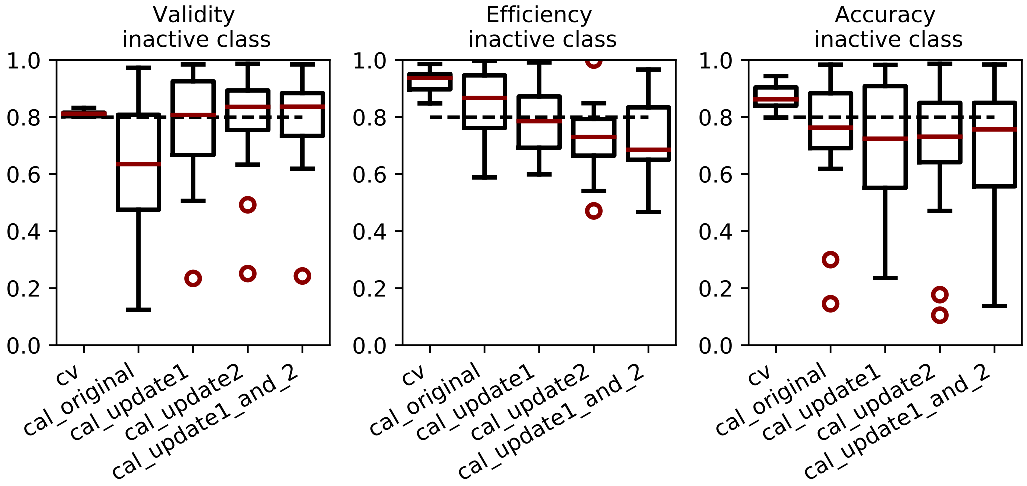

(a) Evaluation for inactive compounds

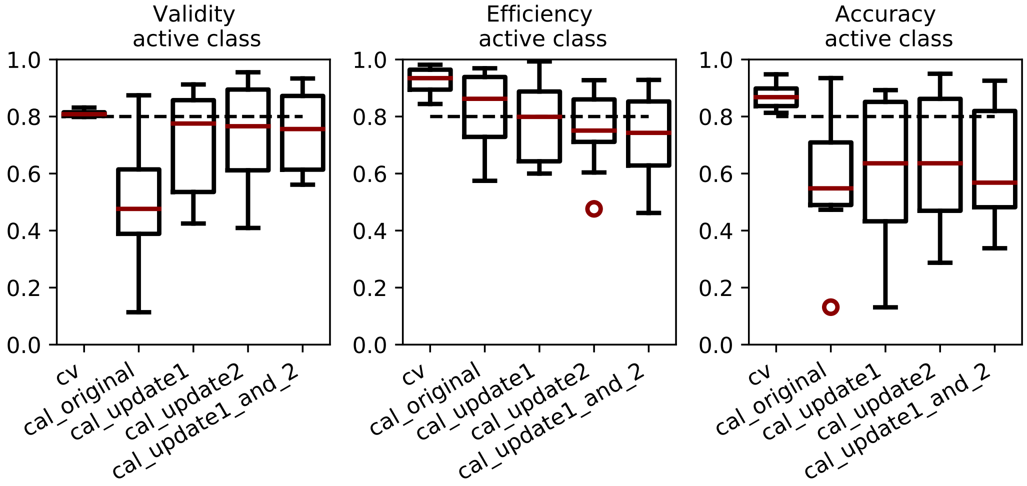

(b) Evaluation for active compounds

Figure S1. Class-wise time split evaluation (validity, efficiency, accuracy) of CV experiments and predictions for the holdout set using the original (cal_original), update1 (cal_update1), update2 (cal_update2) and combined update1_and_2 (cal_update1_and_2) calibration sets for twelve ChEMBL datasets.

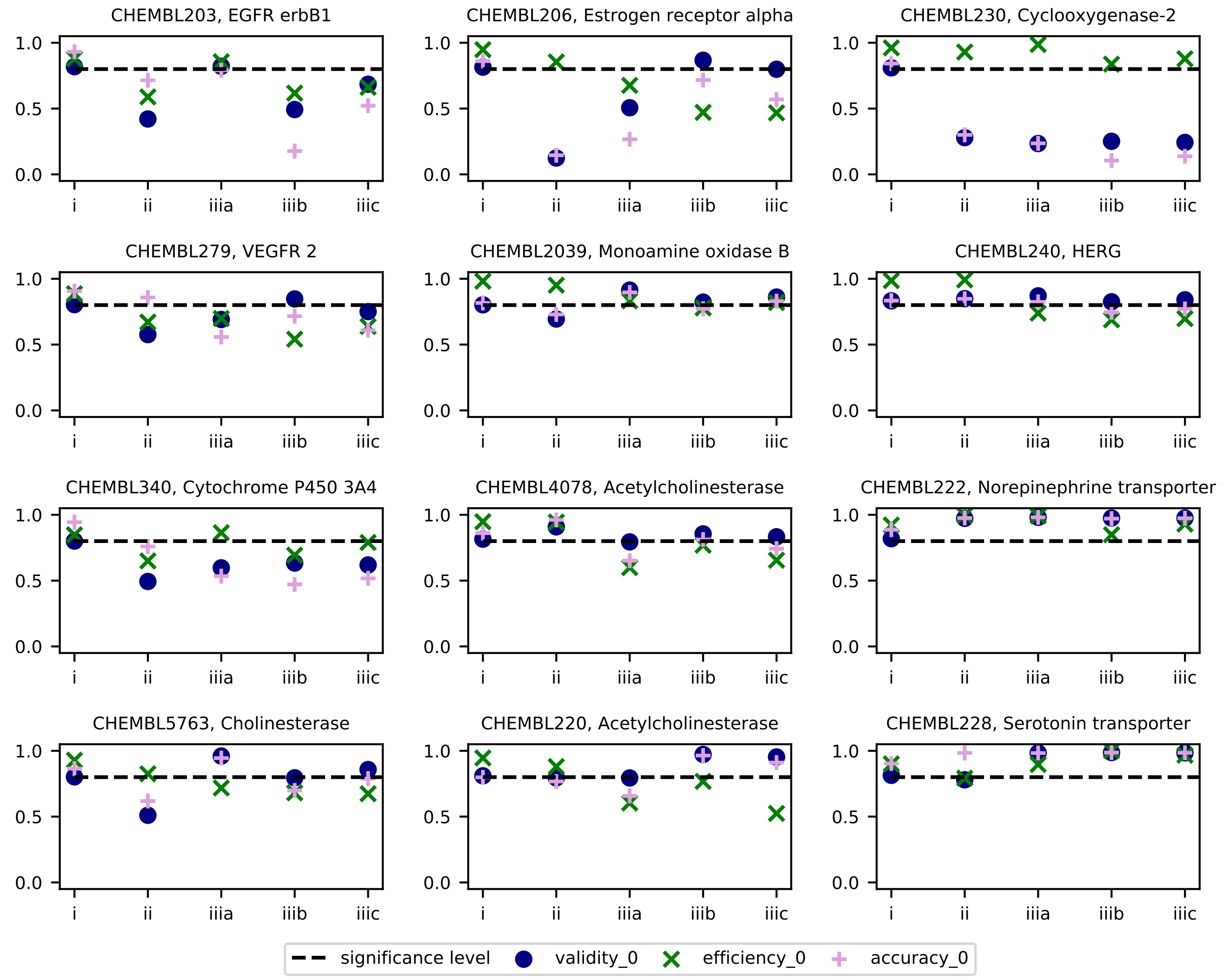

Figure S2. Inactive compounds evaluation of time-split experiments for individual ChEMBL endpoints. i) cross-validation on training data, predict holdout data using ii) original calibration set iiia) update1, iiib) update2, iiic) combined update1+2 calibration sets.

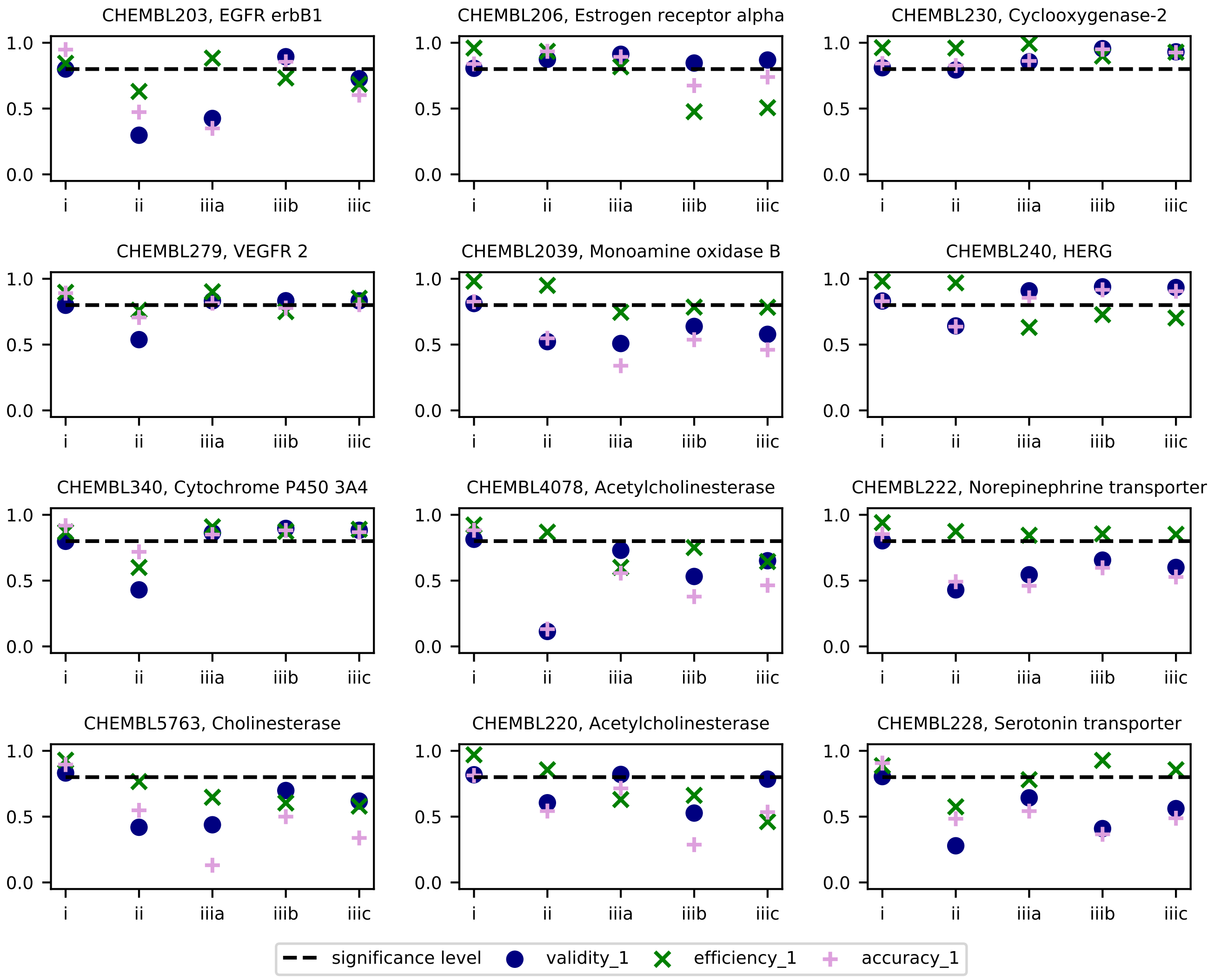

Figure S3. Active compounds evaluation of time-split experiments for individual ChEMBL endpoints. i) cross-validation on training data, predict holdout data using ii) original calibration set iiia) update1, iiib) update2, iiic) combined update1+2 calibration sets.

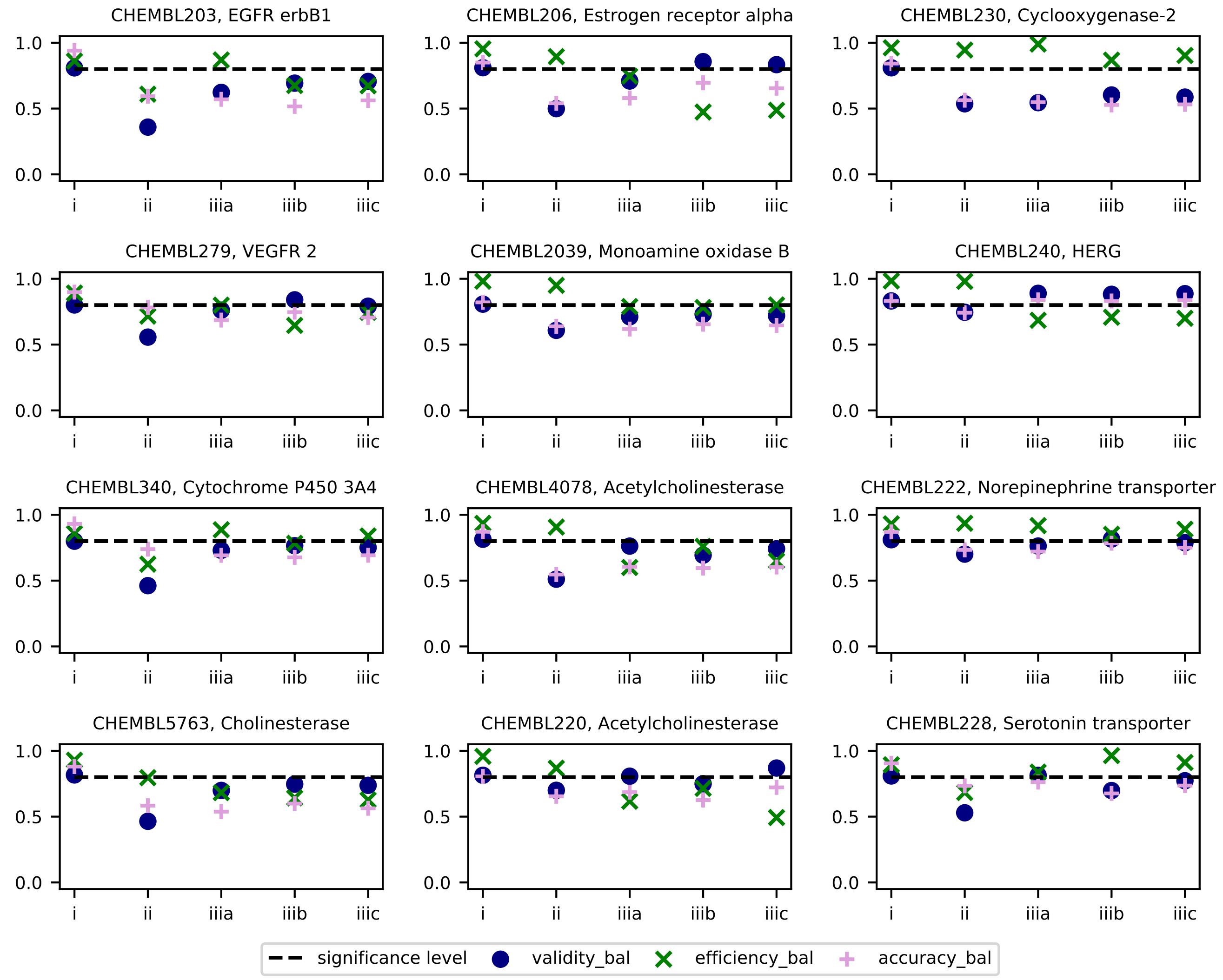

Figure S4. Balanced evaluation of time-split experiments for individual ChEMBL endpoints. i) cross-validation on training data, predict holdout data using ii) original calibration set, iii) updated calibration set, a) update1, b) update2, c) combined update1+2 sets. The doted line at 0.8 denotes the expected validity for the chosen significance level (of 0.2).

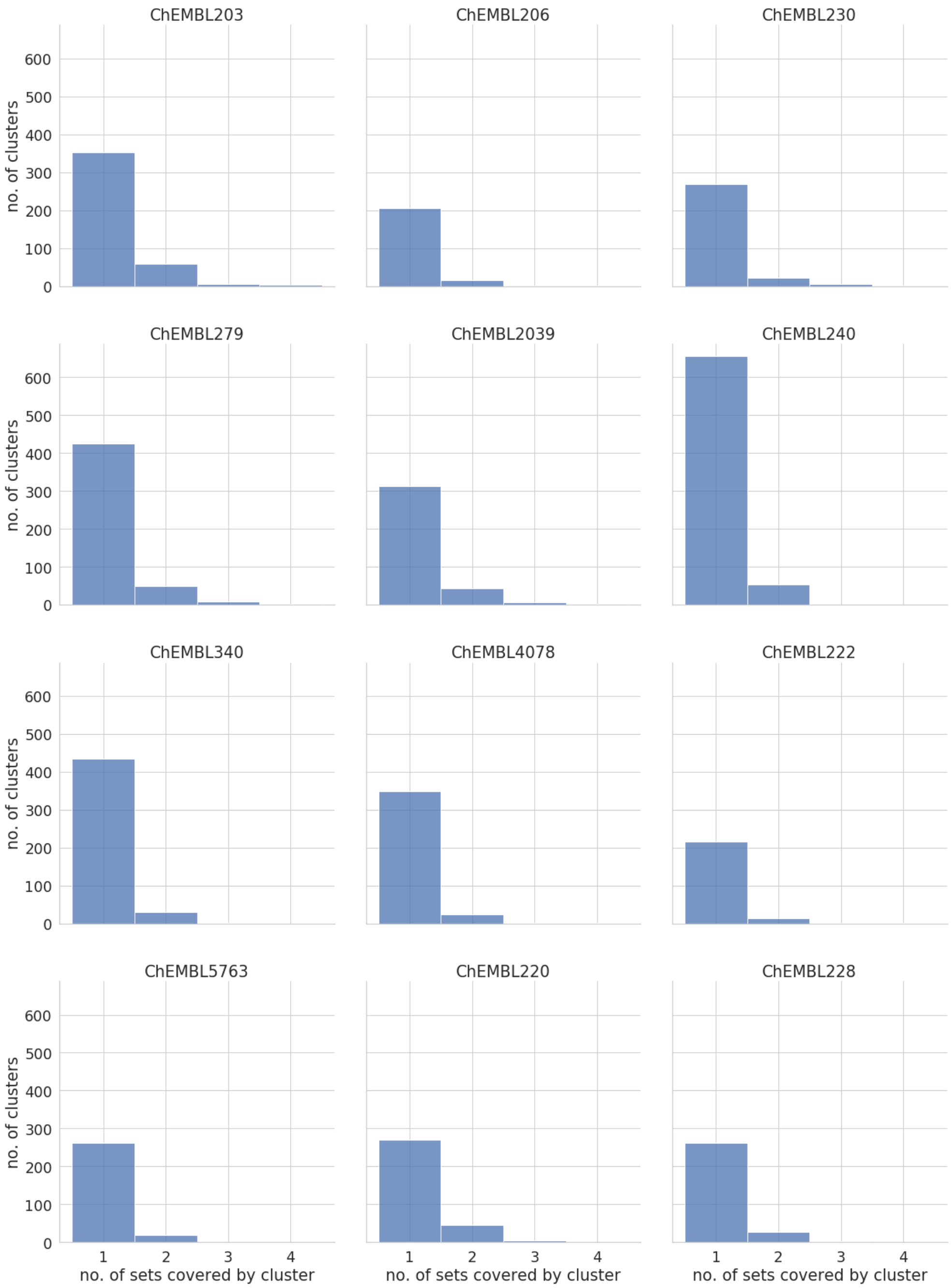

Figure S5. Spreading of clusters amongst the data subsets (i.e. splits) for the ChEMBL datasets. Most of the clusters (with at least two compounds) do not spread over more than one subset (i.e. training, update1, update2 or holdout set).

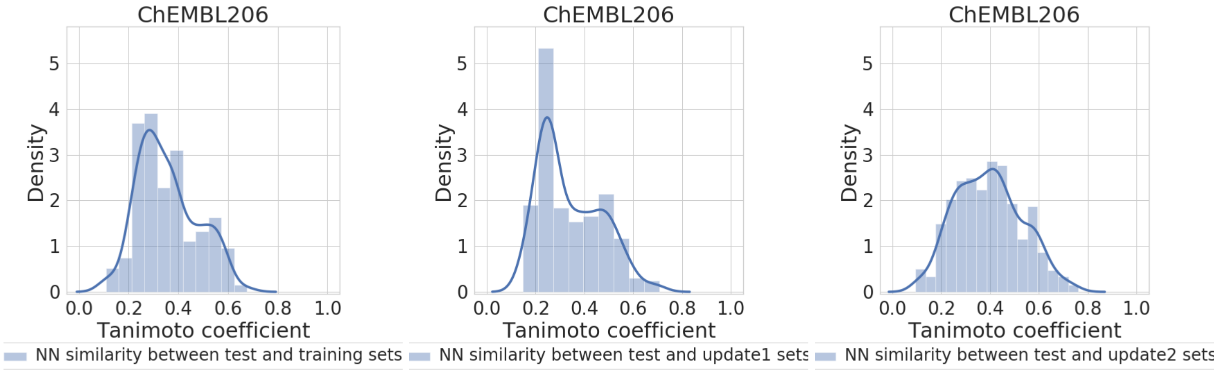

(a) training set **(b)** Update1 set **(c)** Update2 set

Figure S6. Distribution of Tanimoto coefficients between each holdout compound to its nearest neighbour in the corresponding subset (training, update1 and update2) for ChEMBL206 endpoint .

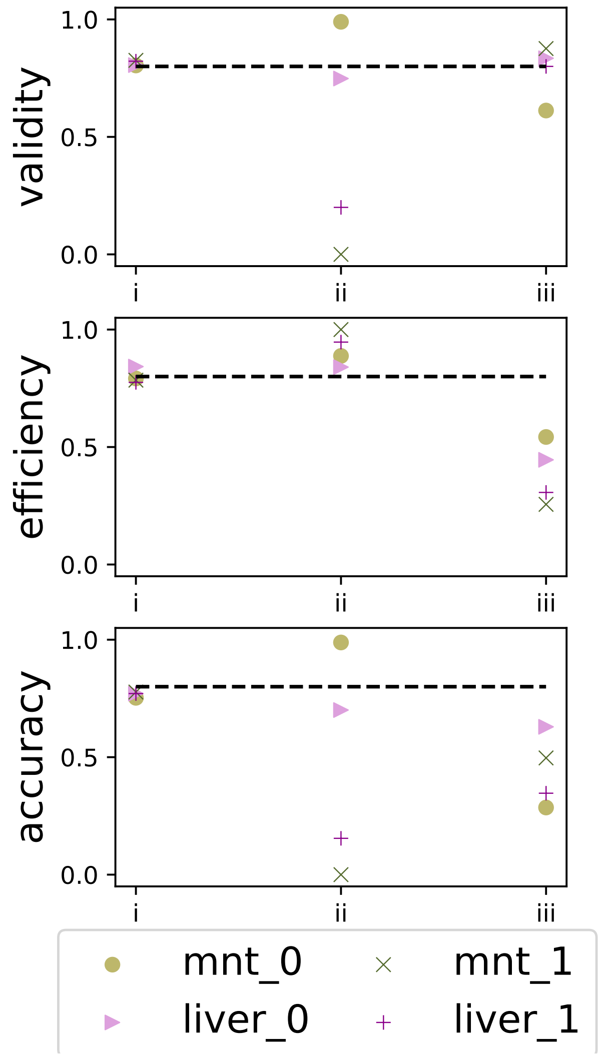

Figure S7. Time split evaluation (validity, efficiency, accuracy) of experiments i) CV, predictions using ii) original calibration set, iii) update calibration set for the liver toxicity and MNT inhouse datasets.

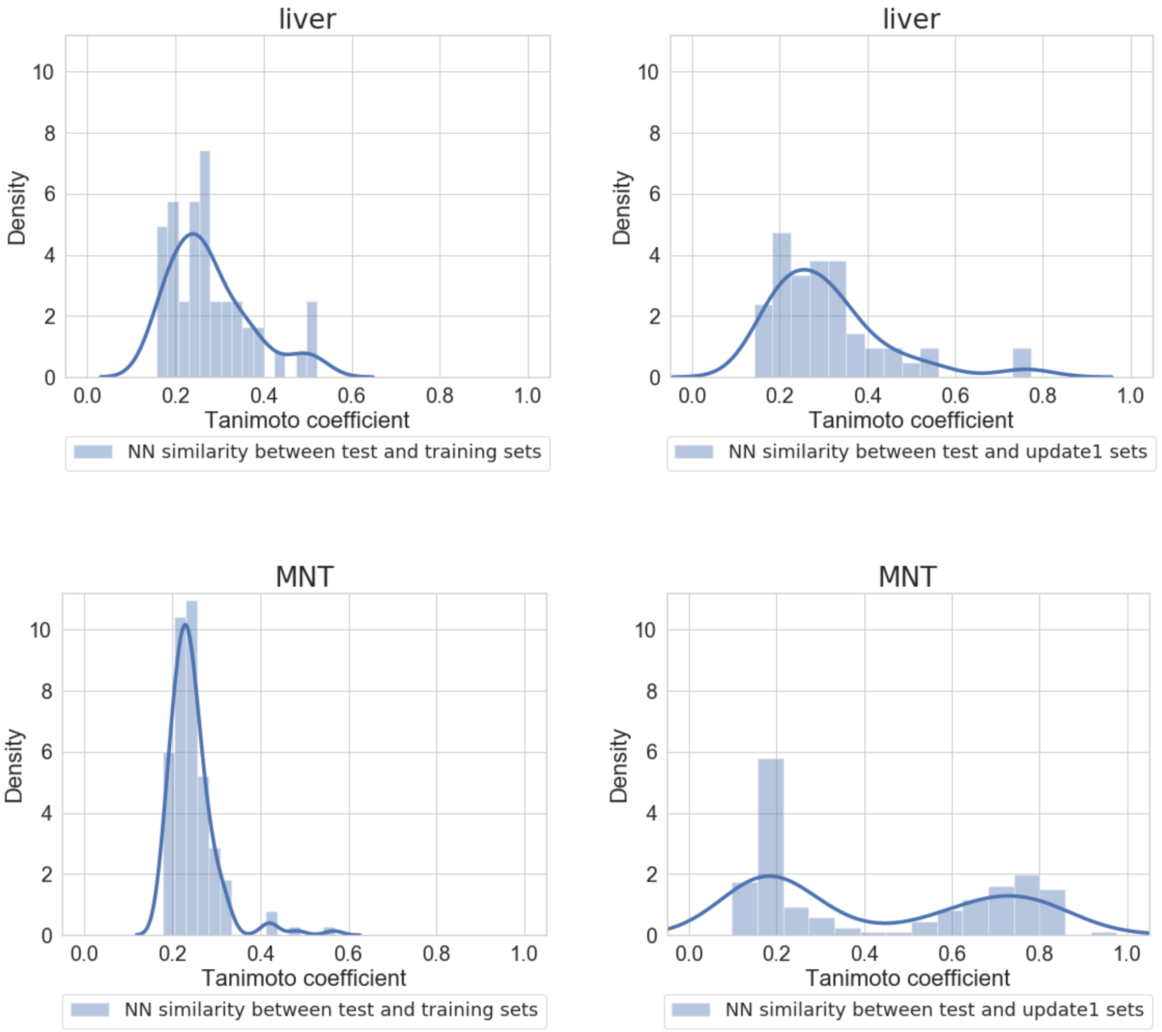

Figure S8. Distribution of Tanimoto coefficients between each holdout compound to its nearest neighbour in the training (left) and update (right) set for the liver (top) and MNT (bottom) endpoints.
